# Determination of the De Novo Minimum Selection Concentration of Trimethoprim In Vivo for *Escherichia coli* Using *Galleria mellonella:* A Pilot Study

**DOI:** 10.3390/microorganisms13010003

**Published:** 2024-12-24

**Authors:** Jaime Knox Macleod, Zina Gestels, Said Abdellati, Thibaut Vanbaelen, Chris Kenyon, Sheeba Santhini Manoharan-Basil

**Affiliations:** 1 STI Unit, Department of Clinical Sciences, Institute of Tropical Medicine, 2000 Antwerp, Belgiumckenyon@itg.be (C.K.); 2 Division of Infectious Diseases and HIV Medicine, University of Cape Town, Cape Town 7700, South Africa

**Keywords:** *Escherichia coli*, antimicrobial consumption, AMR, resistance, trimethoprim, minimum selection concentration, MSC

## Abstract

We investigated whether the maximum residual levels of trimethoprim permitted in food (Acceptable Daily Intake—ADI) could select for de novo trimethoprim resistance in *Escherichia coli* in vivo. We designed chronic infection models of *E. coli* in *Galleria mellonella* and exposed them to sub-ADI doses of trimethoprim through a single-dosing regimen. The emergence of trimethoprim resistance was determined by isolating the target bacteria on selective agar plates, followed by species confirmation using MALDI-TOF mass spectrometry. The minimum inhibitory concentration (MIC) was assessed via the E-test to determine *E. coli* susceptibility to trimethoprim. Notably, exposure to as low as one-tenth of the ADI dose through a single-dosing regimen resulted in the selection of trimethoprim-resistant *E. coli*. Our findings indicate that trimethoprim doses ten-fold lower than the established ADI threshold could induce resistance to trimethoprim in *E. coli*. These results highlight the importance of considering antimicrobial resistance induction as a key factor when determining ADI levels in food.

## 1. Introduction

In recent years, several studies have established that subinhibitory concentrations of antimicrobials can induce de novo antimicrobial resistance (AMR) [1,2,3]. The minimum selection concentration (MSC) is defined as the minimum concentration of an antimicrobial that can select for resistance [1,4]. MSC can be categorized into two types: MSC_select_, which is the lowest concentration of an antimicrobial that selects for a resistant phenotype over a susceptible one [5], and MSC_de novo_, defined as the minimum concentration of an antimicrobial that can induce de novo resistance. Antimicrobials are frequently detected in the environment at sub-MIC concentrations, and numerous studies have found that these low concentrations can select for AMR [4,6,7,8]. For example, Gullberg et al. found that the ciprofloxacin MSC for *Escherichia coli* can be up to 260 times lower than the MIC of the susceptible strain (MIC_susc_) [4]. Another study performed by Gullberg et al. used an *E. coli* plasmid that conferred resistance to a range of antibiotics, including tetracycline and trimethoprim, and found that the multidrug-resistant plasmid was selected for at concentrations far below the MIC_susc_ [1].

An emerging body of research has suggested that numerous foodstuffs contain low concentrations of antimicrobials that are above the MSCs [8,9]. More recently, in vivo models have been developed to directly test if the concentrations of antimicrobials allowed in food could induce AMR [8]. These studies have assessed if the acceptable daily intake (ADI) of an antimicrobial can induce AMR. The ADI is defined by the Food and Agriculture Organization/World Health Organization (FAO/WHO) as “an estimate of the amount of a food additive in food or beverages expressed on a body weight (bw) basis that can be ingested daily over a lifetime without appreciable health risk to the consumer” [10]. Thus far, these studies have found that ciprofloxacin and erythromycin doses as low as 1/10th of the ADI can induce resistance in *Klebsiella pneumoniae* and *Streptococcus pneumoniae* in a *Galleria mellonella* model [8,11]. In the current study, we use the same model, *G. mellonella*, to assess if sub-ADI doses of trimethoprim can induce resistance to trimethoprim in *E. coli*. We chose trimethoprim as it is one of the five most commonly used antibiotics for food animals worldwide [12].

*G. mellonella* is being increasingly used as a model for studying human infections due to several advantages. The larvae possess an innate immune system that shares functional similarities with the mammalian immune system, including cellular and humoral responses [13]. The model is cost-effective, easy to handle, and does not require the ethical considerations associated with vertebrate models. Additionally, *G. mellonella* can be maintained at human body temperature, allowing for the study of pathogens at physiologically relevant conditions [13]. These features make *G. mellonella* a valuable tool for investigating microbial pathogenicity and testing antimicrobial agents.

High concentrations of trimethoprim have been reported in untreated municipal wastewater systems in numerous studies, with levels between 0.17–8.8 µg/L in regions of South Africa [14] and Scandinavia [15] and concentrations as high as 28 µg/L in surface waters in Pakistan [16]. Furthermore, a study in the United Kingdom found that trimethoprim was the second most frequently detected antimicrobial in a range of animal-based food and drink products, with concentrations ranging from 55.2–461.7 µg/kg [17].

The European Medicines Agency (EMA) determines the acceptable daily ingestion (ADI) of a medicinal compound based on studies assessing microbiological and cellular toxicity thresholds [18,19,20,21,22,23]. For antimicrobials, ADIs are predominately derived from microbiological toxicity data, typically established by evaluating the MICs for common human bacterial commensals or pathobionts, such as *E. coli*, and estimating dose exposure levels in the human colon [18,19,20,23]. Notably, the potential for induction of or selection for AMR is not directly included [23]. The ADIs serve as the basis for setting maximum residue limits (MRLs) that represent the maximum concentration of the compound allowed in food products based on the average consumption patterns of those food products [23,24]. According to the latest EMA reports, the ADI for trimethoprim is established at 4.2 μg/kg [25].

Trimethoprim is a synthetic antimicrobial mainly used to treat urinary tract infections (UTIs) [26]. It is also commonly used in human and veterinary medicine, frequently in combination with sulphonamide [25], to treat a range of other infections [27]. It is either bactericidal (in combination with sulphonamide) or bacteriostatic via inhibiting the folic acid synthesis pathway [28] in which the reduction of dihydrofolate to tetrahydrofolate is blocked, causing disordered nucleic acid synthesis [26]. Previous studies have found that mutations at residues P21, A26, D27, L28, W30, I94, and F153 of the dihydrofolate reductase (DHFR) enzyme play an important role in trimethoprim resistance in *E. coli* [29]. DHFR is encoded by *dhfr* genes, including *folA.* Of the emergent mutations, the L28R mutation is the most frequent mutation in the coding region of *folA* [29]. It not only increases the trimethoprim MIC but also acts as a compensatory mutation for the reduced catalytic activity caused by other DHFR mutations [29]. Brolund et al. investigated the distribution of *dfr*-genes and integrons in *E. coli* and found that the prevalence of class I and II integrons was 85% and 57%, respectively [30]. Sequencing analysis revealed *dfrA1*—the most common trimethoprim resistance present together with either *dfrA5*, *dfrA7*, *dfrA14* or *dfrA17* genes [30]. The likelihood of integron carriage increased with the number of resistance determinants [30]. *mgrB*, a gene involved in trimethoprim and colistin resistance [31], was identified by screening the *E. coli* single-gene knockout library [32], and Shi et al. investigated the mechanism of trimethoprim resistance, showing that its deletion upregulated the PhoP/Q system, leading to *folA* overexpression and DHFR-related resistance [33,34]. Mutations in DHFR’s promoter and ribosome-binding site also contributed to trimethoprim resistance [35,36]. Finally, deletion of *glyA*, a gene encoding serine hydroxy methyltransferase from the folate pathway, increased sensitivity to trimethoprim, indicating its implication in trimethoprim resistance [37].

We hypothesized that the EMA ADI dose of trimethoprim could induce resistance in vivo. We tested this hypothesis using a *G. mellonella* model of *E. coli* infection treated with peri-ADI doses of trimethoprim.

## 2. Materials and Methods

### 2.1. Bacterial Strains and Growth Conditions

The strain used in this study was the *E. coli* strain ATCC 25922, commonly used in quality control for antimicrobial susceptibility testing [38] and susceptible to trimethoprim with a MIC of 1 µg/mL [39]. It belongs to serotype O6, biotype 1, and was initially isolated from a clinical sample in Seattle, Washington in 1946 [38]. The assembled genome of this strain is 5.20 Mb, comprising two plasmids (48,488 and 24,185-bp, respectively) and a chromosome (5,130,767-bp) [38]. The MIC for trimethoprim was confirmed as 1 µg/mL using an E-test (AB bioMerieux, Craponne, France) prior to performing the main experiments.

### 2.2. Preparation of Live Microbial Inocula for Infection

*E. coli* strains were cultured from frozen stocks onto BD^TM^ Columbia Agar supplemented with 5% sheep blood for ≤16 h at 37 °C with 5% (*v*/*v*) CO_2_. Single colonies were selected and spread onto fresh agar plates that were incubated at 37 °C with 5% (*v*/*v*) CO_2_ for 6 h. Suspension of *E. coli* was made with phosphate buffer saline (PBS) and then injected into the hemocoel of the *G. mellonella* larva. The dose of *E. coli* was optimized to allow the recovery of the bacteria up to 3 days post-inoculation, minimizing larval mortality.

### 2.3. Injection of G. mellonella Larvae

The study included control groups of 10 larvae and experimental groups with 30 larvae per condition. Healthy, non-discoloured larvae in their last larval stage, weighing 250–450 mg, were selected and placed into sterile petri dishes in groups of 10 per petri dish. These larvae were incubated at 37 °C with 5% (*v*/*v*) CO_2_ throughout the experiment.

In the experimental groups, each larva was injected with 30 µL of bacterial suspension into the last right pro-leg. After 10–20 min, the larvae were injected in the last left pro-leg with various doses of trimethoprim. Injections were administered using 0.3 mL U-100 insulin syringes (BD Micro-Fine, Franklin Lakes, NJ, USA), with one syringe and needle used per petri dish (10 larvae per petri dish).

One control group followed the same procedure as the experimental groups, receiving *E. coli* inoculation in the last right pro-leg followed by 10 µL/larva of phosphate-buffered saline (PBS) in the last left pro-leg (positive control). The other control group received only 10 µL/larva of PBS in the last left pro-leg (negative control).

### 2.4. Concentration of Trimethoprim Injected

As previously mentioned, the EMA ADI for trimethoprim is 4.2 µg/kg bw. Based on this, the equivalent dose of trimethoprim for these experiments was calculated to be 1.57 ng, using the average weight of the *G. mellonella* larvae (380 mg). The doses tested include 15.7 ng (10x ADI), 1.57 ng (ADI), and 0.157 ng (0.1x ADI) per larva.

Upon completion of each experiment, both surviving and dead *G. mellonella* larvae were kept at −80 °C overnight to sedate and euthanize them. Following this, the larvae were autoclaved at 121 °C for 15 min and discarded.

### 2.5. Retrieval of E. coli from G. mellonella

One to 5 larvae from each group of 10 were randomly selected at 24 and 48 h after the injection for the retrieval of *E. coli.* Half of the surviving larvae were used at 24 h, and the remaining live larvae were used at 48 h. The selected larvae were then placed in −80 °C refrigeration for 60 s until no movement could be observed. They were then put into a petri dish for an incision to be made between two segments close to the larval tail using a scalpel. The haemolymph was then squeezed into 1.8 mL centrifuge tubes containing 50 µL PBS, after which the tubes were vortexed. *E. coli* from the *G. mellonella* haemolymph extraction were cultured by plating equal volumes of the extraction onto MacConkey agar with and without trimethoprim. The plates containing trimethoprim were prepared with a concentration of three times their MIC (3 µg/mL trimethoprim).

MacConkey plates were incubated at 37 °C with 5% (*v*/*v*) CO_2_ for 24 h, and the number of *E. coli* colonies that appeared dark blue to violet was counted. Growth was assessed for up to 48 h post-haemolymph extraction. A subset of colonies from the antibiotic plates and a random selection of colonies from the non-antibiotic plates were selected and sub-cultured on MacConkey plates without antibiotic for species identification by MALDI-TOF-MS and for determining the trimethoprim MIC by E-test (AB bioMerieux, France). The E-tests for MIC determination of trimethoprim were performed on Mueller-Hinton agar (MHA) plates and incubated for 18–20 h at 37 °C with a 5% (*v*/*v*) CO_2_. The EUCAST guidelines were followed in defining the trimethoprim resistance as >2 µg/mL for *E. coli* (https://mic.eucast.org/search/, accessed on 3 January 2024). The E-test strips were placed on a plate inoculated with 0.5 McFarland concentration of *E. coli* isolates and read at 100% inhibition of bacterial growth at 18–20 h.

For the stability experiment, trimethoprim-resistant isolates were passaged daily for five consecutive days on a 5% sheep blood BD^TM^ Columbia Agar plate, and MIC was assessed post-passage using E-tests in order to determine if the observed resistance phenotype was stable over time.

### 2.6. MALDI-TOF MS Species Identification

Species identification of the isolates were carried out using Matrix-Assisted Laser Desorption/Ionization-Time-of-Flight mass spectrometry (MALDI-TOF MS) on a MALDI Biotyper^®^ Sirius IVD system using the MBT Compass IVD software and library, version 2023 (Bruker Daltonics, Bremen, Germany). This was done by spreading each bacterial isolate onto a polished steel target plate, covering it with 1 µL of α-cyano-4-hydroxycinnamic acid (CHCA) matrix solution, drying it, and loading the target plate. The results of the identification were classified as either reliable or unreliable based on the recommended cut-off values of 1.7 and 2 for genus and species levels, respectively.

## 3. Results

**Figure 1:**
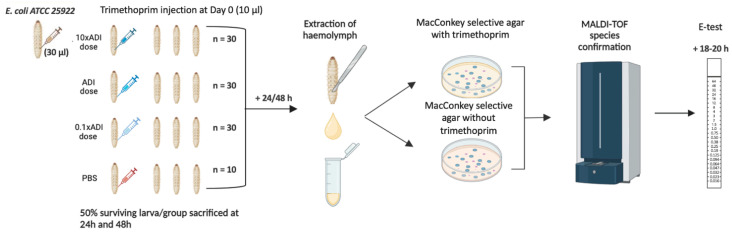
Schematic overview of study methodology (Figure produced with Biorender).

An overview of the study methodology is provided in Figure 1.

### 3.1. Colonization

**Figure 2:**
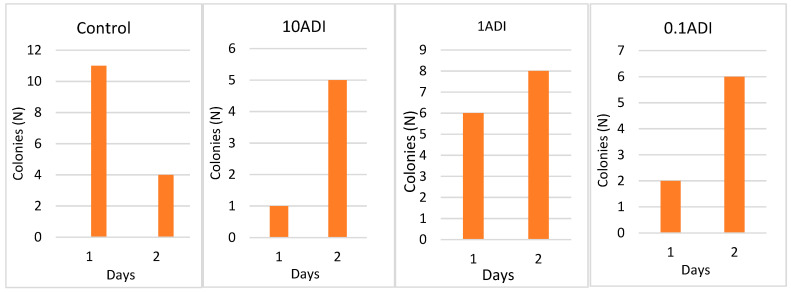
Colonization of *G. mellonella* larvae with *E. coli* up to 2 days post inoculation of *E. coli* and various concentrations of trimethoprim, with the number of colonies (N) observed on plates without trimethoprim.

*E. coli* were successfully recovered on a selective agar plate for up to 2 days following inoculation (Figure 2). Species identities were confirmed through MADLI-TOF MS (Table 1). Each haemolymph extraction was plated in triplicate for each condition across all experiments performed, with 50 µL of haemolymph plated onto two MacConkey agar plates—one with and one without trimethoprim. *E. coli* was isolated on all non-trimethoprim plates following the 2 and 48-h extraction.

### 3.2. Colony Emergence and Identification

No growth was observed on any of the trimethoprim plates after the initial 24 h incubation, but after an additional 24 h incubation, bright pink colonies appeared on all the plates except those from the positive and negative control groups. More specifically, no colonies emerged on the trimethoprim plates from the positive or negative control groups at 24 h or 48 h. The colonies from the trimethoprim plates were all sub-cultured onto MacConkey agar for MIC determination and species identification. All these colonies were identified as *E. coli* via MALDI-TOF (Table 1). 

**Figure 3:**
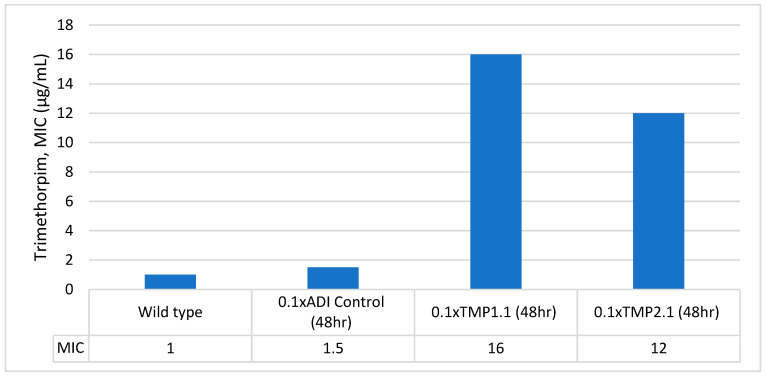
A graph depicting the difference in trimethoprim MICs of the *E. coli* isolates obtained from the baseline-wildtype, 0.1x ADI control, and the resistant isolates.

Two of 6 colonies (2/6) from the 1x ADI group and both (2/2) colonies from the 0.1x ADI group from the 24 h trimethoprim plates along with two of 6 colonies (2/6) from the 0.1x ADI group from the 48 h trimethoprim plate and one from the (1/2) 0.1x ADI positive control samples from both 24 and 48 h were randomly selected for MIC determination via E-test. Elevated trimethoprim MICs were observed for two colonies—0.1x ADI-48h-TMP1.1 (16 µg/mL) and 0.1x ADI-48h-TMP2.1 (12 µg/mL) samples (Figure 3).

### 3.3. Stability of Trimethoprim-Resistant Isolates

Two trimethoprim-resistant isolates (0.1x ADI-48h-TMP1.1 and 0.1x ADI-48h-TMP2.1) along with one isolate from the 0.1x ADI control group were used to assess if the observed increase in MIC was a stable phenotypic change. Only isolates from the 0.1x ADI group were passaged, as this was the lowest concentration that induced resistance.

At day 5, the isolate (0.1x ADI-48h-TMP1.1) with the pre-passage trimethoprim MIC of 16 µg/mL displayed two ellipses in the post-passage E-test, with one reading at 1.5 µg/mL and the other at 12 µg/mL (Supplementary Figure S1). The second isolate (0.1x ADI-48h-TMP2.1) with a pre-passage MIC of 12 µg/mL retained this MIC post-passage. The MIC of the 0.1x ADI control group also remained unchanged at 1 µg/mL after passaging.

## 4. Discussion

In this pilot study, the lowest dose that induces de novo resistance to trimethoprim in *E. coli* in the *G. mellonella* model was found to be 0.157 ng/mL. This concentration is 6369-fold lower than the MIC and 10-fold lower than the EMA ADI for trimethoprim. It is also orders of magnitude greater than the concentration of trimethoprim observed in a range of animal-based food products in the United Kingdom [17]. Forty-eight hours after receiving a single dose of one-tenth of the ADI (0.1x ADI) of trimethoprim, resistant colonies emerged with an increase in MIC of up to 16-fold (MIC-16 µg/mL). These findings suggest that single doses of subinhibitory concentrations of trimethoprim, even 10 times below the acceptable daily intake, can select for de novo resistance in vivo. These results are consistent with other MSC studies involving *E. coli*. For example, a study by Gullberg et al. determined the MSC_de novo_ for ciprofloxacin and tetracycline to be 0.1 ng/mL and 15 ng/mL (4). Another study performed by Gullberg et al. investigated the selective effect of sub-MIC concentrations on multidrug-resistant plasmids and found the MSC of trimethoprim to be 1/6th (33 ng/mL) of the MIC of the susceptible strain (MIC_susc_.) [1]. The MSC_de novo_ was not assessed in this study. One possible explanation for the higher MSC observed in the plasmid study is the higher fitness cost of a resistance mechanism being carried on a plasmid rather than the chromosome [1]. In 2014, Gullberg et al. noted that moving the resistance genes from the plasmid to the chromosome reduces the fitness cost associated with resistance, which in turn reduced the MSC for the tetracycline, trimethoprim, and erythromycin by 2- to 15-fold [1]. Our study’s MSC result for trimethoprim is significantly lower than either of these estimates, which may be due to chromosomal mutations with little to no fitness costs, differences in the *E. coli* strain used, or related to peculiarities of the *G. mellonella* infection model.

Earlier, it was assumed that the mutant selective window (MSW) lies between the MIC_susc_ and the MIC_res_ and that sub-MIC concentrations of antibiotics did not confer a selection pressure [40]. However, results from this study, along with previous MSC studies, have established that the sub-MIC selective window is significantly wider and extends to the MSC. Sub-MIC concentrations can select for resistant strains or induce de novo resistance [4]. Long-term persistence of resistance due to low concentrations of antibiotics in the environment may be sufficient to maintain resistance in a population of bacteria for bacterial pathogens whose life cycle involves periodic growth in the environment, such as *E. coli* [4]. The initial fitness costs associated with resistance mutations are often compensated for by secondary mutations, which allows these resistance mutations to spread in various settings [41].

In our trimethoprim experiments, an emergent trimethoprim-resistant isolate (MIC of 16 µg/mL) exhibited a double ellipse after 5 days of in vitro passaging. One ellipse was at 1.5 µg/mL and the other at 12 µg/mL, which is suggestive of a heterogenous subpopulation, whereby the population of *E. coli* with the MIC of 16 µg/mL was more dominant pre-passage, potentially due to low-cost or cost-free mutations as a result of the antibiotic selective pressure. However, post-passage, the population with a MIC of 12 µg/mL was more fit without the selective pressure and, therefore, became more dominant. Such heterogeneous subpopulations are sometimes observed in gradient tests as a presence of bacterial colonies within the growth inhibition zone (ellipse) [42], as observed in our experiment.

A major study limitation is that we did not conduct whole genome sequencing (WGS) to evaluate emergent resistance-associated mutations. We only used MALDI-TOFF to identify the isolates with trimethoprim resistance. Without WGS we cannot exclude the possibility that these isolates were preexisting strains of trimethoprim resistant *E. coli* from the *G. mellonella*’s endogenous microbiota. The fact that no *E. coli* isolates with trimethoprim resistance were detected in the control group does however make this explanation unlikely. We also only evaluated the effect of trimethoprim on one strain of one bacterial species. Furthermore, this ATCC 25922 strain is used mainly for quality control purposes. It would, therefore, be useful to include other bacterial species and other strains of *E. coli* that are more pathogenic or clinically relevant in future experiments. It would be particularly important to include other commensal bacterial species, such as commensal *Neisseria* spp., which, by virtue of their high prevalence, would be most exposed to antimicrobials in food [43]. Our model relied on establishing a chronic haemolymph infection in *G. mellonella* to determine whether low doses of trimethoprim could induce AMR. A more applicable approach would be to examine if these low doses ingested orally could induce AMR in bacteria in humans or other mammals [44]. Moreover, our study only explored the effect of low antibiotic doses on the emergence of de novo resistance without assessing the potential enrichment of pre-existing resistant strains or the spread of AMR through mobile genetic elements [1,5]. Future studies should investigate whether these low doses could interact with other substances in food, such as heavy metals, to induce and select for AMR as has been established in vitro [45]. Long-term, daily dosing regimens should also be implemented to evaluate if this would have a more pronounced effect on the emergence of AMR. These limitations mean that this study should best be considered as a pilot study that requires confirmation in a larger study that is able to address these limitations.

Despite these limitations, this pilot study is the first of its kind to assess if low doses of trimethoprim can select for AMR in vivo. The positive findings from this study, together with those of previous similar studies, suggest the need for equivalent studies in mammals [8,11,46]. Finally, our results should encourage the relevant authorities to include MSCs of antimicrobials in their determination of ADIs and MRLs. While the *G. mellonella* model is useful for testing numerous bug-drug combinations, further validation in mouse and human models will be required to validate the findings.

## Figures and Tables

**Table 1:** Phenotype and MALDI-TOF species identification with the respective confidence scores for each sample from MacConkey plates. The samples are named according to plate of colony, for example, 0.1x-24h-TMP1.1 denotes the trimethoprim plate and colony ID (plate 1, colony 1 = 1.1) for the 0.1x ADI condition from the 24 h extraction.

Sample ID (MacConkey)	Phenotype	Detected Species	MALDI-TOF Confidence Score	MIC (µg/mL)
10x-24h-TMP4.1	Pink	*Escherichia coli*	2.46	8
1x-24h-TMP1.1	Pink	*Escherichia coli*	2.07	1.5
1x-24h-TMP2.1	Pink	*Escherichia coli*	2.01	1
1x-24h-TMP3.1	Pink	*Escherichia coli*	2.2	1.5
0.1x-24h-TMP1.1	Pink	*Escherichia coli*	2.01	1.5
10x-48h-TMP1.2	Pink	*Escherichia coli*	1.8	1.5
10x-48h-TMP2.1	Pink	*Escherichia coli*	2.21	1.5
1x-48h-TMP1.1	Pink	*Escherichia coli*	1.8	1.5
1x-48h-TMP2.1	Pink	*Escherichia coli*	2.02	1.5
0.1x-48h-TMP1.1	Pink	*Escherichia coli*	1.92	16
0.1x-48h-TMP1.2	Pink	*Escherichia coli*	1.91	1
0.1x-48h-TMP1.3	Pink	*Escherichia coli*	2.09	1.5
0.1x-48h-TMP2.1	Pink	*Escherichia coli*	1.92	12

## Data Availability

The original contributions presented in the study are included in the article/Supplementary Materials, further inquiries can be directed to the corresponding author.

## Supplementary Materials

The following supporting information can be downloaded at: https://www.mdpi.com/article/10.3390/microorganisms13010003/s1, Figure S1: A photograph depicting the double ellipse observed in the E-test for isolate 0.1x ADI 1.1 (48 h) 5 days post in vitro passaging.
