# Supplementary material for: Determination of the De Novo Minimum Selection Concentration of Trimethoprim In Vivo for Escherichia coli Using Galleria mellonella: A Pilot Study
Source: Microorganisms. 2024 Dec 24;13(1):3. doi: 10.3390/microorganisms13010003 (PMC11767374; doi:10.3390/microorganisms13010003)
Supplement: Supplementary file 1 [file microorganisms-13-00003-s001.zip › microorganisms-3310058-supplementary.pptx]

## Slide 1
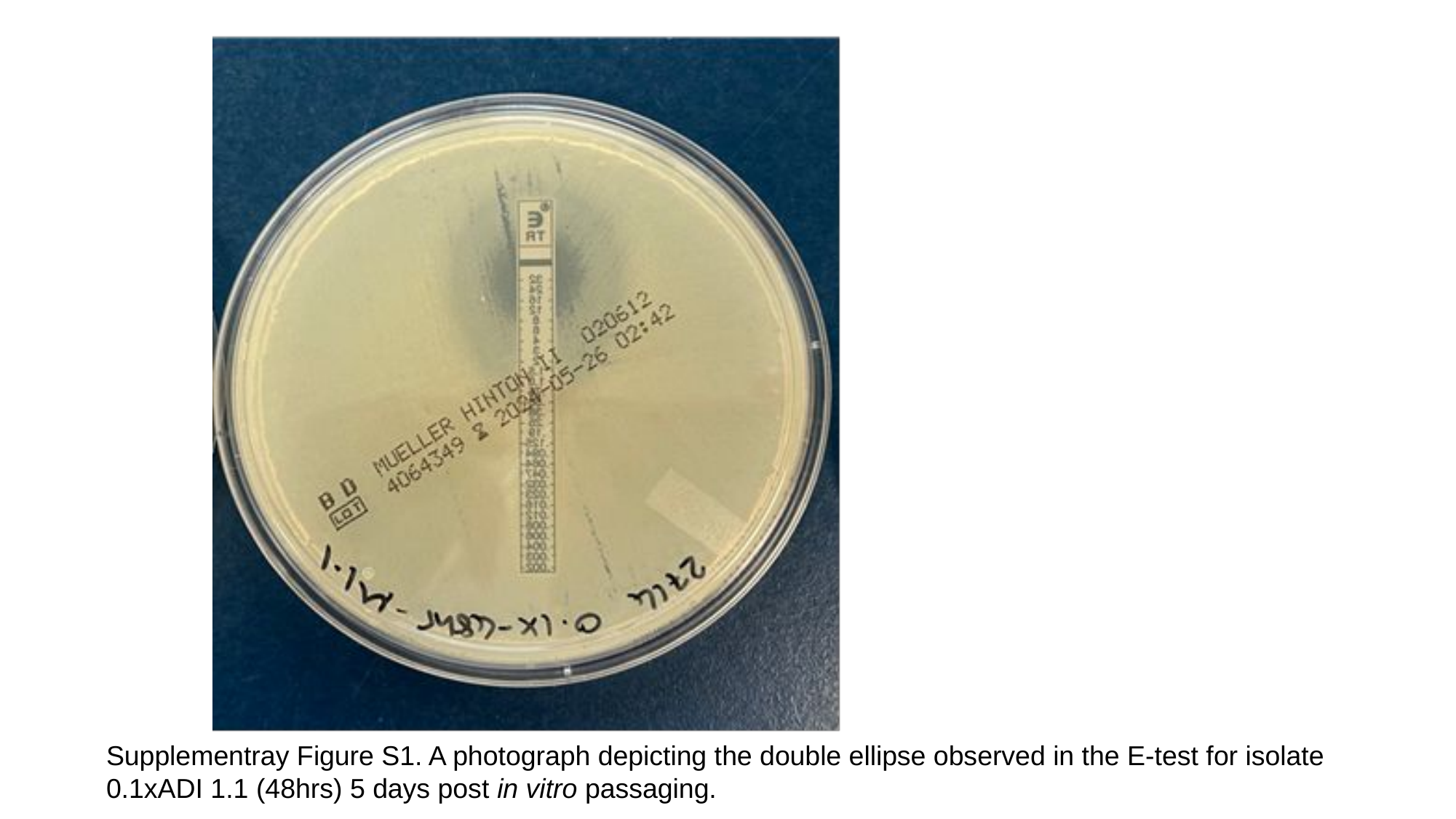

Supplementray Figure S1. A photograph depicting the double ellipse observed in the E-test for isolate 0.1xADI 1.1 (48hrs) 5 days post in vitro passaging.
